# Caregiver experiences of public services following child trauma exposure: a qualitative study

**DOI:** 10.1186/s13033-018-0190-6

**Published:** 2018-04-10

**Authors:** Victoria Williamson, Sarah L. Halligan, Bronwyne Coetzee, Ian Butler, Mark Tomlinson, Sarah Skeen, Jackie Stewart

**Affiliations:** 1 Department of Psychology, University of Bath, Bath, UK; 2 Department of Psychiatry and Mental Health, University of Cape Town, Cape Town, South Africa; 3 Department of Psychology, Stellenbosch University, Stellenbosch, South Africa; 4 Department of Humanities and Social Sciences, University of Bath, Bath, UK; 5 KCMHR, Institute of Psychiatry, Psychology and Neuroscience, King’s College London, London, SE5 9RJ UK

**Keywords:** Child, Trauma, Parent, Public services, Hospital, Police, Judicial system

## Abstract

**Background:**

Many children in low and middle income countries (LMIC) are exposed to trauma. Contact with public services are a potential influence on parent–child reactions and coping post-trauma. Little is known about how caregivers perceive these interactions.

**Methods:**

The aim of this study was to explore caregivers’ experiences of accessing and interacting with public services post-trauma and perceptions of needed improvements to public services in a LMIC context. Qualitative interviews were conducted with 20 female caregivers from a high-risk settlement in South Africa after child trauma exposure.

**Results:**

Three themes and seven sub-themes were identified regarding caregivers’ perceptions of interactions with public services post-trauma. The key themes identified related to (1) communication and exchanges with law enforcement, (2) consequences of an under-resourced justice system and (3) importance of communication and empathy in the healthcare system. Interactions with police were often positive. However, caregivers explained that police-family communication post-trauma could be improved and may help to lessen caregiver anxiety and concerns for the child’s safety post-trauma. Caregivers perceived the judicial system to be under-resourced as contact with the judicial system was often protracted and caused child anxiety and distress. Medical treatment was reportedly rushed, with extensive waiting times and little information provided to caregivers regarding the child’s injuries or treatment. Some medical staff were perceived as unsympathetic during the child’s treatment which was found to exacerbate caregiver and child distress post-trauma.

**Conclusions:**

This study provides insight into caregiver experiences of accessing public services following child trauma exposure in a high-risk LMIC context. Public services were perceived as oversubscribed and under-resourced and negative interactions often influenced caregiver responses and appraisals of child safety. Given the impact of poor interactions with public services on families post-trauma, additional research is needed to investigate feasible improvements to public services in LMIC.

**Electronic supplementary material:**

The online version of this article (10.1186/s13033-018-0190-6) contains supplementary material, which is available to authorized users.

## Background

Exposure to traumatic events during childhood, including road traffic accidents or acute medical emergencies, can lead to the development of psychological adjustment difficulties. Children in many low and middle income countries (LMIC) are exposed to multiple traumatic events as a result of several factors, including community violence, extreme poverty, child labour and internal displacement [19]. In South Africa, rates of child trauma exposure have been found to be extremely high [11, 30, 31]. For example, 39% of girls in South Africa report experiencing some form of sexual violence under the age of 18 years, 48% of children have been exposed to severe community violence, and 15% report neglect [3, 29]. Parents of children exposed to trauma in Khayelitsha (a peri-urban settlement in South Africa) have been found to report significant anxiety for their child’s physical safety and feel helpless to prevent future trauma [38].

Following child trauma exposure, families are often in contact with medical, police, or social services. Contact with such services can have a substantial effect on parent–child reactions and coping post-trauma [21]. For example, recent qualitative research in a relatively low-risk, Western context found parental perceptions of problematic medical care and a lack of information regarding child recovery in emergency departments (EDs) to contribute to parental perceptions of the child as vulnerable, resulting in overprotective parenting responses [36]. The impact of child trauma exposure can potentially be mitigated by trauma-informed care delivery by services that are prevention oriented and focused on improving the mental health functioning of children and their families [21]. However, little is known about families’ experiences of interacting with services post-trauma in high-risk, LMIC contexts. Generally, public services, including the police, ambulance services and hospitals, in South Africa are over-subscribed and under-resourced [15, 20, 29]. Previous research has found community members report negative experiences of emergency service personnel (e.g., police, ambulance services) following traumatic events and often perceive personnel to be doing an inadequate job to support and protect the community [7, 33]. First responders and health care providers in EDs are pivotally placed to prevent persistent stress reactions in children following trauma [18, 21]. An in-depth exploration of families’ experiences of interacting with public services following child trauma exposure in a high-risk, LMIC context may provide insight into families’ support needs and have implications for improvements to clinical practice and policing. Therefore, the objective of this study was to explore families’ experiences of accessing formal support following child trauma exposure, interactions with public services, and caregivers’ perceptions of needed improvements to public services.

## Methods

This study received approval from the Stellenbosch University Health Research Ethics Committee (N14/08/112) and the University of Bath Ethics Committee (15-022).

### Participants

Participants were 20 female primary caregivers whose children, aged 6–16 years, were exposed to a traumatic event meeting DSM-5 criterion A [1] in the last 2 years. Participants were all residents of Khayelitsha, a large peri-urban township in Cape Town, South Africa. Exclusion criteria included: caregiver being unaware of the child’s trauma; existing organic brain damage or intellectual disability in the child precluding mainstream schooling; the child being orphaned by the traumatic event; child registered with child protection; and concerns that the respondent caregiver was the perpetrator of the trauma. Twenty-five caregivers were approached to take part in the study and the five who did not participate either did not have time to take part or became uncontactable.

Opportunity sampling and snowball sampling methods were used to recruit participants. Further details regarding participant recruitment and informed consent procedures are described by Williamson et al. [38]. Data from this sample has been previously reported in a study investigating parental responses following child trauma exposure [38].

### Assessments

Face-to-face, semi-structured qualitative interviews were conducted with participants by bilingual data collectors. All data collectors had extensive experience in both quantitative and qualitative data collection.

Data collectors received additional training in qualitative interview methods, interviewing trauma exposed individuals and clinical risk and referral procedures. An experienced qualitative researcher with a background in trauma research (JS) provided the training. Data collector training included sessions on the nature, function and conduct of qualitative research methods, the potential impact of traumatic events on individuals, in-depth discussions regarding the objective of each interview question, and mock interviews and feedback from the broader research team. Detailed feedback on interview content was provided at weekly supervision meetings, and the quality of participant interviews was assessed throughout data collection.

Interviews were conducted between February and August 2015. Interviews lasted approximately 1 h 45 min and were audio-recorded. Caregivers were given a R120 (approximately USD 8.23) voucher for their participation in the study, the standard amount required by Stellenbosch University Health Research Ethics Committee. Following the interview, caregivers were given the chance to ask any additional questions they had, and were offered the opportunity to receive a referral letter to local mental health services for themselves or their child if desired.

#### Semi-structured interview

The interview guide was developed based on the study research questions and the literature of parent–child experiences following child trauma exposure. Two formative focus groups were conducted with local community members to ensure interview questions were appropriate given the Xhosa sociocultural context. Interview questions focused primarily on caregiver experiences following child trauma exposure; however, data were also collected regarding caregiver perceptions of the formal support available from public services, and views of (need for) support and barriers to formal support. Interviews were audio-recorded with participant consent. Interviews were conducted in Xhosa, the primary local language. To ensure accuracy, transcripts underwent a three-part translation and transcription process. Interviews were first translated and transcribed by a bilingual transcriber who did not conduct the interview. The transcripts were then reviewed by the data collector who conducted the interview for accuracy. Any disagreements in the translation of the transcripts were discussed and resolved following a re-examination of the interview audio-recording.

### Analysis

Transcripts were analysed independently by two of the authors (VW and BC), using Nvivo (VW) and ATLAS.ti v 7 (BC) (http://www.atlas.ti.com). Using a six phase approach, consistent with thematic analysis as laid out by Braun and Clarke [8], each of the authors (1) read and re-read transcripts in order become familiar with the data, (2) selected and coded relevant extracts from the transcripts, (3) selected similar codes and grouped them into themes, (4) organised codes together in line with conceptual and theoretical similarities, (5) refined each theme and allocated suitable theme names, and (6) presented the results of the data analysis. In order to ensure consistency of coding between the two authors, regular meetings were held to discuss progress and to discuss any discrepancies in the codes. We used a process of peer debriefing to ensure the credibility and trustworthiness of the findings [35]. As part of the peer debriefing process, feedback was solicited from the data collectors who conducted the interviews to ensure the codes and themes accurately reflected the sociocultural context of participants. Moreover, feedback regarding interpretation of the data was regularly sought from co-authors SH and MT who have experience with child psychopathology research and qualitative methods. Reflective memos were written throughout data analysis to record early interpretations of the data and relationships between concepts [4, 35]. A reflexive journal was also kept during data collection and analysis by the primary researcher (VW) for reflexivity.

## Results

### Sample characteristics

Of the 20 caregivers, 17 were mothers (*M *= 41.3 years, range 29–57 years) with low income and employment levels (see Additional file 1: Table S1 for full demographic details). Eleven of the participating children were female, with a mean age of 11.5 years (range 6–16 years). On average, children were reportedly exposed to three traumatic events in the last 2 years. Index traumas included: sexual assault (20%), physical assault (25%), road traffic accidents (20%), witnessing a sudden death of a close friend or family member (15%), and witnessing or experiencing community violence (20%). Seven (35%) participants reported contact with the judicial system, 16 (80%) had contact with a hospital and 14 (70%) reported contact with the police.

### Themes

Three key themes were identified specifically relating to caregivers experiences of and interactions with public services following child trauma exposure (see Table 1). Findings are illustrated with anonymised excerpts with pseudonyms assigned to all children and caregivers to ensure confidentiality.

**Table 1:** Themes and sub-themes following thematic analysis

Theme and sub-themes
Communication and exchanges with law enforcement
Problematic communication
Less involvement with greater perceived threat
Vigilante justice
Consequences of an under-resourced justice system
Protracted court procedures
Judicial system ill-equipped for disability
Importance of communication and empathy in the healthcare system
Impact of communication from healthcare staff
Uncaring, prolonged and chaotic medical care

### Communication and exchanges with law enforcement

#### Problematic communication

Caregivers often reported significant communication difficulties with the police following child trauma exposure. If the event was reported to the police, caregivers received little information regarding the progress of the criminal investigation. Furthermore, perpetrators were frequently released, either on bail or without charges, back into the community without prior notice given to the child or their family. This experience of limited communication is illustrated in the excerpt below.

*Interviewer: Up until now how far are the police with the case?*

*Mother: There is nothing concrete. We only receive texts from them saying the case was [posted] to a certain investigator. They do that all the time, nothing straight…It would be nice when the police say we have caught the criminals…that can give us hope that the police are doing their job. When they are quiet that does not give me hope that they are doing their work.*

Poor police communication with families contributed to caregiver perceptions of the police as ineffectual and caused significant caregiver distress and anxiety for their child’s safety. The release of perpetrators into the local community was also thought to prevent children from forgetting the trauma as children often, inevitably, came into contact with them.

#### Less involvement with greater perceived threat

Contact with the police following the child’s trauma exposure and caregiver’s perceptions of their interactions with the police varied depending on the type of trauma experienced by the child. For example, in cases of child exposure to gang violence, caregivers perceived the police to be unable to offer much support as they were overwhelmed by the problem and able to offer only limited protection. For example, one mother stated:

*Mother: That is why I say we feel safe when the police are around but when [the gangsters] do not listen to them, we lose hope because those [gangsters] are going to do what they want to do. If they want to kill you, they will kill you and there is no hope. Even the police have realised that they are only listened to when they come about eight or nine vans not two vans.*

This perception of the police having limited effectiveness in responding to and preventing community violence reportedly contributed to caregivers’ acute anxiety for their child’s physical safety post-trauma. Caregiver anxiety led to the use of protective strategies to ensure child safety, such as increased monitoring of the child’s whereabouts or sending the child to live with relatives in rural villages which were considered safer. In some cases, interactions with the police post-trauma were considered potentially dangerous. In particular, caregivers and children were concerned that being seen to report events to the police may lead to further victimisation if the perpetrators were not apprehended. One participant recalled:

*Interviewer: I heard you saying that the police came and took the children to identify the criminals, what was happening in your mind?*

*Mother: Yoooh that bothered me, I was not ok with [it at] all, we even said… what if the children get into trouble for going to identify the criminals… the police parked their police van in front of our house…we will not know whether the person doing this to us is not watching…I told the police that they should never come to our house with their van… I was afraid that maybe the criminals might see [our children with the police] and say those children are from that house and make a follow up and maybe come to our house again.*

#### Vigilante justice

In response to poor experiences of policing, several caregivers and community members resorted to vigilante justice following the child’s traumatic event, with alleged perpetrators beaten for retribution and to deter further criminal behaviour.

*Mother: I did not involve the Police since those children were under age they were going to be arrested and be released soon. Since we knew them…I organised for them to be collected on Sunday…we found them in their homes and I punished them myself… they were taken and beaten…I wanted to do it myself, I wished I could stab them where they stabbed my son.*

However, in some instances, caregivers attempted to resolve disputes that arose with the perpetrator of the child’s trauma informally using non-violent means. If the conflict could not be adequately resolved, caregivers reported contacting the police for support. Police involvement in these instances was experienced as helpful as they acted as mediators and provided the caregiver with advice, as seen in the following quote:

*Mother: The other [woman] was a sibling to the guy who wanted to rape my child and [she] is rude and she drinks… when I went there with the police she was insulting me but the police said I must not answer her, I must just keep quiet.*

### Consequences of an under-resourced justice system

#### Protracted court procedures

Caregivers reported that court procedures and dates were often postponed (especially in instances of lost dockets) and required multiple visits, which proved to be overwhelming both physically and financially.

*Mother: In another month we would be called twice in a week, or maybe we would go three or four times in a month as time went by we would go once a month even to the social workers we would go on a month.*

Court involvement was problematic for young school children, as it was not only emotionally and psychologically burdensome to repeatedly confront the traumatic events, but also deprived children of time at school. Further, the court experience was described as lonely for the caregiver and their child as they often attended the proceedings alone without family and friends.

#### Judicial system ill-equipped for disability

Although this only pertained to one child, it is important to highlight the apparent lack of resources available in the judicial system to deal timeously and effectively with children who have disabilities. In addition to regular postponement and delay, the court proceedings for this child were further put on hold in order to seek out and appoint a sign language interpreter to communicate with her. The mother recalls:

*Mother: Last year [in] October we were called and the child was taken to counselling, she was given dolls there to show what happened and she did, she showed them and they said they will call us when they have a person who can do the sign language.*

### Importance of communication and empathy in the healthcare system

Following child trauma exposure, medical treatment was sought from government run hospitals, community health centres, or specialist rape crisis centres. Caregivers reported that rape crisis facilities provided access to psychological support services needed to deal with the traumatic events.

*Mother: …at least there is light now because when you are in such a situation you can go to clinics now and be sent to counsellors and receive counselling and make you forget what had happen.*

#### Impact of communication from healthcare staff

Interactions with medical staff following the traumatic event varied. However, in several cases, failure on the part of medical staff to communicate with caregivers about their child’s injuries and the next steps left impressions of neglect and disinterest. Caregivers perceived nurses in particular to be unsympathetic towards them which in some cases forced caregivers to seek healthcare services at other facilities which were usually situated further from their homes. As stated by one mother:

*Mother: That is why I was hurt by the way we were received [at the hospital] but that did not pull me down. I took the next step and went to [a different hospital].*

Clear communication with children and their caregivers was experienced as helpful in reducing the anxiety that was often experienced in trauma and emergency care units of clinics and hospitals. Positive interactions with medical staff, including being received in a friendly, professional manner and exhibiting genuine concern for the child, were deemed by caregivers as necessary and important in providing psychological and emotional comfort to children in the early hours post-trauma. As reported by one caregiver:

*Aunt: Immediately when we arrived she was taken to her ward and the doctor explained to us that they are going to do 1,2,3, I was told to come back at a certain time and that she was going to be taken to theatre at a certain time. When I arrived the following day her operation was done. Even when I visit, her doctor would come and explain to me her progress, how she was last night, or if there were complications, new changes, the doctor would tell me.*

#### Uncaring, prolonged and chaotic medical care

Caregivers often perceived their child to have received poor care that was disorganised in nature, with children abruptly transferred to different hospitals. This experience of chaotic medical care is illustrated in the following excerpt:

*Mother: No [his wound] was dressed in [one hospital], then they transferred him to [a different hospital], the new hospital…and when he arrived there it was found out that a pipe inserted in him that helped with blood circulation was wrongly inserted so they had to take it out and reinsert it.*

Procedurally, transfers to different hospitals require referral letters from the clinics in their catchment area prior to providing the necessary care. In instances where caregivers had received poor reception from the clinic, they would arrive at the hospital without referral letters. Failure to produce referral letters meant further prolonging of care and exacerbated caregivers’ anxiety, frustration and negative perceptions of healthcare services. One mother recalled:

*Mother: It is not the same … they will have to know how you came there, what happened, the nature of the problem, maybe your problem is not the problem that is supposed to be referred there, it is a minor problem, it was supposed to be done [somewhere else]. You will struggle like that because if it’s a minor problem they will know and tell you it was supposed to be done there.*

Despite the urgency with which many caregivers sought attention for their children (many of whom were in significant pain) at the health care facilities following the traumatic event, they reported experiencing considerable waiting times. Caregivers were very distressed following their child’s trauma upon arrival at health care facilities and the lengthy waiting times experienced often exacerbated caregivers’ feelings of anxiety and helplessness, as well as concerns for their child’s physical well-being post-trauma

*Mother: I was hurt because when you take a person to the clinic you expect to be helped hence they say we should take them to the place of help when you cannot manage to help them yourself. If I was able to help him I would have not wasted my money and take him to the clinic.*

## Discussion

The purpose of this study was to explore and understand families’ experiences of accessing formal support and interacting with public services following child trauma. Our results demonstrated three public service interactions that children and their caregivers report involvement with following a trauma, namely (1) interactions with the police, (2) interactions with hospitals and emergency departments, and (3) interactions with the judicial system. The results indicated that during contact with each of these organisations families are usually confronted with poor service with little hope of foreseeable improvements in the near future.

A key theme emerging from the data related to caregiver interactions with the police post-trauma. Caregivers perceived the police as overwhelmed and ineffective in managing the high levels of community violence. This often contributed to caregiver anxiety and concerns for their child’s safety, leading to the use of somewhat excessive caregiver strategies to ensure child safety, including vigilantism, changes to the child’s routine and increased monitoring. Caregivers’ implementation of such socio-environmental changes on the child may potentially have negative implications for child adjustment post-trauma by increasing the child’s perceived vulnerability to threat (e.g., [5, 16, 37]). Overall, the poor perceptions of the police in being unable to manage high levels of community violence in this sample had a significant impact on caregiver responses post-trauma, which may have deleterious effects for both child and family coping.

Caregivers also highlighted the poor communication from the police following child trauma exposure, with families given no notice regarding the release of perpetrators or the progress of their case. Effective communication with caregivers by the police may benefit families as the provision of accurate information delivered in a clear, sensitive manner by police has been reported to be helpful to parents following child trauma exposure [28]. Moreover, caregivers reported concerns that being seen to report the event to the police may cause them to be re-targeted by the un-apprehended perpetrators. While it is unclear how often the retargeting of victims and their families occurs due to lack of available data, in order to improve the experiences of children and families following child trauma exposure in high-risk contexts, law enforcement ought to be sensitive to the safety and contextual issues underlying caregivers help seeking needs. Additional research is needed to investigate more effective ways for the police to communicate with and provide support to families in LMIC settings following child trauma exposure with the aim of improving the caregiver-child experience. It is possible that interventions used in other LMIC contexts, such as Latin America may be beneficial. Top-down interventions focusing on institutional strengthening (e.g., judicial, police and penal systems) and programs to improve community-policing relations have been found to be effective in reducing crime as well as improving community confidence in the police [26]. Additional research is needed to evaluate whether such violence reduction interventions would be feasible and effective in this context.

In instances where the child’s trauma resulted in a court case, many caregivers reported heavily protracted court proceedings requiring multiple visits. Extended delays were reported in a case where the child had disabilities, which highlights the limited legal system resources in this context [13]. Child involvement in legal proceedings following trauma exposure has been found to be particularly stressful and confusing for children, with children often misunderstanding court proceedings [14]. Previous research has found that children to experience significant anxiety when recounting their traumatic experience in court [2] and interventions which focus on providing children with support and education about court proceedings have been found to reduce system-induced child stress and anxiety in lower-risk, Western contexts (e.g., [27]). In South Africa, the Children’s Act emphasises the need to create supportive and child-friendly judicial environments for children and their families [10]. However, no such support was routinely available to study families which is notable given the considerable post-trauma distress experienced by the children in the present study (see [38]). These findings suggest a need for more sensitive court support and preparation, including the assessment of child needs post-trauma.

Another major theme related to caregivers’ experiences of healthcare and interactions with medical staff following child trauma exposure. Caregivers reported that their interactions with staff were often rushed, with limited information provided regarding the child’s physical injuries or medical treatment. Child medical treatment was often perceived to be disorganised, with children facing extensive waiting times for care or abruptly transferred to different hospitals. Caregivers also described unsympathetic treatment from medical staff, particularly from nurses, during the course of the child’s treatment and such interactions often exacerbated the distress and anxiety experienced by caregivers and child post-trauma. It is likely that such treatment is a result of the desensitisation of nurses and medical staff due to overcrowding and lack of hospital resources which are commonly reported in South Africa [22]. This experience of medical treatment post-trauma, particularly the lack of information regarding diagnosis or treatment and the limited support from healthcare providers, may contribute to child and caregiver illness uncertainty [25]. High levels of uncertainty of illness can lead to maladaptive patient outcomes and poor coping, as well as caregiver depression and anxiety [23, 25, 34]. Furthermore, previous research has found a perceived lack of information regarding child recovery from medical professionals to heighten parental anxiety for child well-being and contribute to overprotective parental responses [32, 36] which may have negative implications for child adjustment [5, 37]. Providing clear information to caregivers in hospital regarding child treatment and waiting times has been found to be associated with a reduction in parental stress and greater satisfaction with medical care [6, 12, 24]. This approach may be particularly beneficial in LMIC settings and additional research exploring feasible means of implementation is needed.

This study has several limitations. First, only female caregivers were recruited to this study, consistent with the socio-cultural context of female relatives providing the majority of child care [9]. However, male caregivers experiences of accessing and interacting with formal support services may provide valuable insight and should be examined in future research. Second, although considerable efforts were made to ensure data analysis was consistent with the socio-economic and cultural context, we were unable to conduct respondent validation in the present study. Third, qualitative interviews were conducted only with caregivers and future research should include child informants as this may offer useful information about children’s experiences of engaging with public services following trauma exposure. Finally, it is possible that bias may have been introduced as the interviews were conducted in Xhosa and translated to English; however, several steps were taken to ensure data integrity and credibility.

Despite these limitations, the present study expands on the extremely limited research of the experience and challenges faced by caregivers in accessing formal support from public services following child trauma exposure in an exceedingly high-risk context. As LMIC are often characterised by a lack of public sector resources and violent crime, including interpersonal and collective violence [17], the results of this study may be applicable to other high-risk, LMIC contexts. This study highlights the limited attempt by public services to deliver support and treatment to families that is sensitive to their emotional and/or physical needs following child trauma exposure. Additional research is needed to investigate feasible improvements to public services in LMIC to improve the child and family experience post-trauma.

## Additional file

**Additional file 1: Table S1.** Participant Demographic Information.
