# Supplementary material for: Caregiver experiences of public services following child trauma exposure: a qualitative study
Source: Int J Ment Health Syst. 2018 Apr 10;12:15. doi: 10.1186/s13033-018-0190-6 (PMC5894141; doi:10.1186/s13033-018-0190-6)
Supplement: Supplementary file 1 — Additional file 1: Table S1. Participant Demographic Information. [file 13033_2018_190_MOESM1_ESM.docx]

Additional Table S1

*Participant Demographic Information*

| Demographic characteristics | | Mean/Proportion |
| --- | --- | --- |
| Caregiver mean age | | 41.25 (8.02 SD) |
| Caregiver relationship to the child | |  |
| Mother | 85% | |
| Aunt | 10% | |
| Grandmother | 5% | |
| Caregiver marital status | |  |
| Single | | 40% |
| Married / living with partner | | 40% |
| Divorced/Separated/Widowed | | 20% |
| Time since trauma (months) | | 13.85 (8.08 SD) |
| Income | |  |
| 0-1000R | | 15% |
| 10001-2000R | | 25% |
| 2001-5000R | | 40% |
| More than 5000R | | 15% |

*Note:* The Rand-Pound Sterling exchange rate as of 29^th^ April 2017, 500ZAR=£28.88, 1000ZAR= £57.75, 5000ZAR=£288.77. The minimum living wage in South Africa is industry specific; for example, the Ministry of Labour has set the minimum living wage for farm labourers at 2274ZAR per month (£131.33). SD = standard deviation.
